# Differential Effect of Chronic Morphine on Neuronal Degeneration in Male vs. Female Mice

**DOI:** 10.3390/pathophysiology31010012

**Published:** 2024-03-06

**Authors:** Chet Brazile, Ruping Fan, Beau Benoit, Thomas Arnold, Nadejda Korneeva

**Affiliations:** 1 School of Medicine, LSU Health Shreveport, Shreveport, LA 71103, USA; cbr004@lsuhs.edu; 2 Department of Emergency Medicine, LSU Health Shreveport, Shreveport, LA 71103, USA; ruping.fan@lsuhs.edu (R.F.); thomas.arnold@lsuhs.edu (T.A.); 3 Department of Pharmacology, Toxicology and Neuroscience, LSU Health Shreveport, Shreveport, LA 71103, USA; beauxbeno19@gmail.com

**Keywords:** antinociception, demyelination, female, male, morphine, neurodegeneration, opioid, tolerance

## Abstract

Opioid abuse in the United States has been increasing at an alarming rate over the past 20 years. Sex differences are documented for the rates of opioid-related overdoses, abuse patterns, and drug-induced physiological effects. In our previous study, we demonstrated that chronic oxycodone administration in young female rats is associated with neurodegeneration in the brain. Males and females are susceptible to neurodegenerative diseases via differing mechanisms. To investigate whether opioid exposure affects males and females differently, we treated young mice with chronic morphine. We observed that females had stronger antinociceptive responses to acute morphine and showed a delayed development of tolerance. Males had a higher basal Bax level in the brain that correlated with a higher number of apoptotic cells. Morphine increased Bax levels in both males and females without affecting the numbers of apoptotic cells. Morphine increased activated caspase 3 in axons and increased the MBP level in plasma only in females, suggesting a demyelination process. Our data suggest that males are protected from demyelination by having a higher basal BDNF level. Altogether, our results suggest that males and females have different molecular signaling underlying their patterns in the development of morphine tolerance and drug-induced neuronal degeneration.

## 1. Introduction

Opioid abuse in the United States has increased at an alarming rate, with mortality rising by 312% and 596% in males and females, respectively, between 1999 and 2016 [1]. Multiple studies have shown that female used opioids more frequently and for a longer period of time than males, and are more susceptible to drug abuse [2,3,4]. Women are more likely to misuse prescription opioids to treat other problems besides pain, like anxiety, domestic abuse, and self-esteem [2,5]. An analysis of polysubstance use patterns among people who misuse prescription opioids revealed that 37% of them also use alcohol and 15% use alcohol, marijuana, and “other” drugs [6]. Women also experience more severe adverse opioid side-effects than men, with greater sedative and respiratory depression responses, nausea, and dysphoria [7]. A recent report documented sex-specific differences in substance use disorders [8]. Specific psychiatric, psychological, and hormonal factors affect the patterns of drug abuse in women. It was demonstrated that women develop stronger cravings for opioids than men do due to the effect of the sex hormone estradiol activating rewarding signaling in the brain [9]; furthermore, women relapses at higher rates than men [10]. With sex differences appearing in not only opioid-related deaths but also abuse patterns and physiological effects, understanding the reason behind these sex differences could help medical professionals better treat those with an opioid addiction and prevent any further adverse health effects from prescription opioids.

The neurotoxic effects associated with chronic opioid administration have been documented in several human and animal studies. Demyelination and lesions in white matter have been reported for patients using heroin [11,12], methadone [13,14,15], and oxycodone [16]. It was suggested that due to the effect on oligodendrocyte proliferation and maturation, opioids could disrupt the myelination process in the brain [17]. There are reports describing the development of Parkinsonism after the intravenous administration of synthetic heroin [18]. In addition, the induction of apoptosis in neuronal cells and neuronal inflammation were reported after chronic opioid administration in several animal models [19,20,21,22,23,24]. In our previous study, we demonstrated that chronic oxycodone administration in young female rats is associated with several neurodegenerative patterns in the brain: increased oxidative stress, astrocyte activation, decrease and disorganization in white matter, loss of myelin basic protein (MBP), and accumulation of amyloid precursor protein beta [25,26]. Similarly, a recent study showed that female rats with high alcohol preference became dependent on morphine, which was associated with increased oxidative stress, axonal demyelination in the prefrontal cortex, and glial striatal neuroinflammation [27].

Male and female brains are susceptible to neurodegenerative diseases, such as Parkinson’s disease (PD), Alzheimer’s disease (AD), Huntington’s, and multiple sclerosis (MS), via differing mechanisms [28]. It was reported that males are more likely to be affected by PD and females more likely to be affected by dementia, AD, and demyelinating CNS diseases [29,30]. To investigate whether chronic opioid exposure affects males and females differently, we treated young adult mice with 15 mg/kg morphine daily for 30 days and monitored the level of biomarkers associated with neuronal degeneration. Our study revealed that female mice were more sensitive to acute morphine administration and have delayed tolerance development compared to male mice. We observed that the basal level of apoptotic cell numbers was slightly higher in male compared to female brains. Although morphine increased Bax-positive signal, it did not increase the number of pro-apoptotic cells in both male and female mouse brains. Morphine induced activated caspase 3 in axons and increased MBP levels in plasma only in female mice, suggesting a demyelination process in female brains. Interestingly, male mice had higher basal BDNF levels in the brain and plasma, suggesting a protecting mechanism against opioid-induced demyelination. Our results of the different effects of opioids on male and female mice emphasize the importance of including male and female subjects in drug abuse studies.

## 2. Materials and Methods

### 2.1. Animal Model

Male and female C57Bl/6 mice were purchased from Harlan Indianapolis, IN, USA. The animals were housed in groups of four to five animals in plastic cages containing hardwood bedding cages and allowed to acclimatize for at least one week before the experiments were conducted. The mice were kept at a stable temperature (22 ± 1 °C), humidity (40–50%), and 12 hr light/dark cycle throughout the studies. Food (Teklad, Harlan) and water were supplied ad libitum. The protocol for animal studies #P-22-004 was approved by the Louisiana State University Health Science Center, Institutional Animal Care and Use Committee. Two-to-three-month-old male and female mice were assigned to one of two groups (*n* = 5/group for each sex group) receiving intraperitoneal (i.p.) injections of either 15 mg/kg pharmaceutical-grade morphine (cat#M8777, Sigma Aldrich, St. Louis, MO, USA) or its vehicle saline (sterile 0.9% NaCl; cat#76081-514, VWR, RMBIO, Inc., West Jordan, UT, USA). The morphine-treated group was compared directly to the saline-treated control group, which was handled, treated, and sacrificed at the same time and under the same conditions. To monitor the toxicity of morphine, the mice were weighed daily. We did not observe significant weight loss in either group of animals. The effect of morphine on animal behavior was measured by using hot plate (HP), warm water tail-immersion (TIT), open field (OF), and rotarod (RtR) tests.

### 2.2. Behavior Tests

*The hot plate assay* (HP) is a specific test that measures supra-spinal and peripheral analgesia. In the HP test, the animal was placed on a heated at 53 °C plate with the temperature controlled by the UGO Basile 7280 Hot Plate instrument setting. The baseline of response was measured one week prior to the morphine or vehicle injection. The antinociceptive effect of 15 mg/kg of morphine administration was assessed by recording the latency to jump or lick the paws when the mouse was placed on a hot plate (cut-off time 30 s). There were two daily HP tests: one test performed on each mouse prior to and another after 30 min of morphine or saline administration.

*The tail-immersion test* (TIT) is a specific test that measures spinal analgesia. Each mouse was placed in a mouse holder with its distal half of the protruding tail immersed in a conical tube containing warm water (49 °C). The time until withdrawal of the tail was measured (the cutoff time was 15 s). The baseline of response was measured one week prior to the morphine or vehicle injection. There were two daily TIT tests: one test performed on each mouse prior to and another after 30 min of morphine or saline administration. The mice were tested by the HP or TIT assays on alternative days. Data were expressed as a percentage of the maximal possible effect (%MPE) calculated as follows: %MPE = (post-morphine time − base-line time)/(cut-off time − base-line time).

*The open field* (OF) test measures locomotor activity in animals. We assessed the mice on locomotor response using automated activity chambers (Med Associates, Burlington, VT, USA). Behavioral assessments were performed prior to the initiation of the drug treatment (day 0) and then on the 1st, 5th, and 9th days of 15 mg/kg morphine or saline administration. The mice were placed in the chambers for a 20 min session on each of the four days after morphine or vehicle injections prior to the hot plate or tail-immersion tests.

*The rotarod* (RtR) test measures motor coordination in animals. The mice were trained to walk on a rod rotating at 5 rpm for 60 s prior to any drug administration. During the test, the mice were placed on a rod, which accelerated from 4 to 40 rpm in 300 s. The latency to fall and the speed at fall were monitored. We performed the rotarod tests for three days prior of the morphine or saline treatment and then on days 1–3, 27–29, and 58–60 of the 15 mg/kg morphine/saline administration.

### 2.3. Tissue Preparation

*Brain isolation.* Four to five animals from each treatment group were anesthetized with isoflurane. The depth of anesthesia was judged by the absence of a withdrawal reflex when the foot was firmly pinched with forceps. Once deep anesthesia was attained, the mouse was pinned in dorsal recumbency and blood was collected from the heart. The mouse was perfused with 50 mL ice-cold saline through the aortic arch and then with 50 mL ice cold 4% paraformaldehyde in 0.1 M sodium phosphate buffer, pH 7.4 (PB). The mouse brain was kept in 4% paraformaldehyde in PB for 48 h at 4 °C, then in 50% ethanol for 24 h, and finally stored in fresh 50% ethanol at 4 °C until further processing.

*Plasma preparation.* Mouse blood was collected into Eppendorf tubes rinsed with and containing ice-cold 50 μL [50 mM EDTA, 0.9% NaCl] solution. The tube with blood was inverted 10 times and placed on ice until it was centrifuged at 1200× *g* for 15 min at 4 °C. The plasma was collected from the top of the sedimented cells, aliquoted, and stored at −80 °C until further processing. The protein concentrations of the plasma samples were measured by using the Micro BCA™ Protein Assay kit (cat#PI23235, ThermoFisher Scientific, Pierce Biotechnology, Rockford, IL, USA) according to the manufacturer’s protocol.

### 2.4. Immunohistochemical Analysis

Mouse brains were embedded in paraffin and processed, as is described in [26]. Briefly, paraffin-embedded brains were cut into 10 μm thick slices and then mounted on glass slides (Millennia 1000; cat# M1000W, StatLab, McKinney, TX, USA). After deparaffinization and antigen retrieval, each brain section was incubated with the corresponding primary antibodies against Bax (1:4000; cat#50599-2-Ig, VWR), cleaved caspase 3 (1:100; cat#9664, Cell Signaling Technology, Danvers, MA, USA), MBP (1:400; cat#sc-13914R, Santa Cruz Biotechnology, Inc., Dallas, TX, USA), BDNF (1:1000; cat# sc-546, Santa Cruz Biotechnology, Inc.), and testosterone (1:500; cat# MA5-14715, ThermoFisher Scientific) diluted in SignalStain^®^ Antibody diluent (cat#8112, Cell Signaling Technology) and then with secondary antibodies, VectaStain^®^Elite^®^ ABC-HRP kit (cat#PK-6101, Vector Laboratories, Inc., Newark, CA, USA) and with the substrate, DAB–nickel kit (Vector Laboratories, Inc.). The nuclei were visualized by staining with Hematoxylin Gill’s 3 formulation (RICCA Chemical Company, Arlington, TX, USA) and washing in 0.2% ammonia water. The brain slices were covered with HistoChoice^®^ Mounting Media (VWR) and coverslips were analyzed by using an Olympus IX51 microscope with a 40× lens (400× magnification). The images were analyzed by using ImageJ (FIJI) software (https://imagej.net/ij/; accessed 10 January 2022), employing “mean density” or “number of cells” values.

### 2.5. TUNEL Detection

The TUNEL (TdT-mediated dUTP nick end labeling) assay that detects fragmented DNA in cells undergoing apoptosis was performed using the TUNEL detection kit (NeuroTACS II in Situ Apoptosis Detection Kit, Trevigen, R&D Systems, Inc., Minneapolis, MN, USA) according to the manufacturer’s protocol, as is described in [26]. In detail, 10 μm thick slices of mouse brain were permeabilized by incubation with NeuroPore solution™ for 30 min at RT. The positive control slides containing brain sections from saline- or morphine-treated animals were incubated with TACS-Nuclease solution™ added to the labelling reaction mixture. A brown nucleus represented TUNEL-positive cells undergoing apoptosis. The images were taken using an Olympus IX51light microscope. Cells from three fields for each brain region from two animals per treatment (*n* = 6) were investigated. The data are presented as a percentage of TUNEL-positive cells in mouse brain tissues, mean value ± SD.

### 2.6. ELISA Analysis of Plasma Samples

Additionally, 100 μL of plasma sample, diluted to 10 μg/mL in PBS, was dropped into a well of the NUNC MaxiSorb ELISA uncoated plate (cat#10761-500, VWR) in triplicate for each antibody detection, and was incubated overnight slowly rocking at 4 °C. The wells for the negative control contained 100 μL 10 μg/mL BSA (cat#97061-416, VWR). The next morning, the wells were incubated with the blocking solution [0.1% BSA in PBS] for 1 h at RT and then with primary antibodies against GAPDH (1:5000; cat# 10R-G109A, Fitzgerald Industries Intnl., Inc., Acton, USA), MBP (1:500; cat#sc-13914, Santa Cruz Biotechnology), and BDNF (1:500) diluted with blocking solution containing 0.1% Tween-20 for 2 h at RT. The wells were then incubated for 1 h at RT with secondary antibodies: anti-rabbit HRP (1:2000; cat#PI-1000, Vector Laboratory, Inc., Newark, CA, USA) to detect MBP; anti-goat (1:2000; cat#sc-2768, Santa Cruz Biotechnology) to detect MBP; and anti-mouse HRP (1:2000; cat#PI-2000, Vector Laboratory, Inc.) to detect GAPDH. The signal was developed with the ReadiUse™ TMB Substrate Solution (cat#11012, AAT Bioquest, Inc., Pleasanton, CA, USA) and stopped with 0.5 N H_2_SO_4_. The signals were detected at 450 nm. For the data analysis, the BSA negative control was subtracted from the experimental signal. The MBP and BDNF signals were normalized to the GAPDH signal in the corresponding samples. The testosterone ELISA assay was performed using a Testosterone ELISA kit (cat#ADI0900-065, Enzo Life Sciences, Inc., Farmingdale, NY, USA) according to the manufacturer’s protocol. The assays were repeated two times using 30-day plasma samples from five saline-, and five morphine-treated female mice and from three saline- and three morphine-treated male mice.

### 2.7. Statistical Analysis

The results of the experiments are presented as the mean of several independent experiments ± SD or SEM as indicated. Statistical significance was determined by using the Student’s *t*-test, and data with *p* value lower than 0.05 were considered to be statistically different.

## 3. Results

### 3.1. Behavioral Response to Morphine: Female Mice Have Higher Acute Responses to Morphine and Delayed Developments of Tolerance

**Figure 1:**
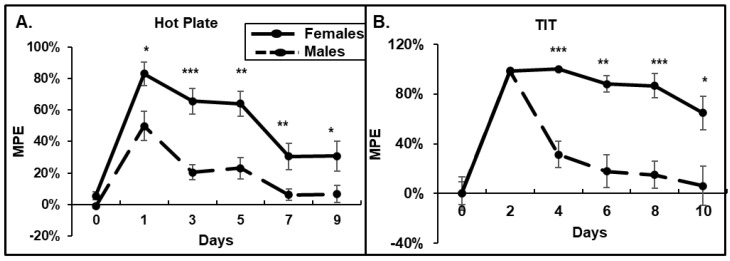
Females have higher responses to acute morphine and delayed developments of tolerance. (**A**) Hot plate assay. (**B**) Warm water tail-immersion test. Twenty male and ten female mice received daily i.p. injections of 15 mg/kg morphine for 30 days. The paw withdrawal latency in the HP and tail flick in the TIT tests were determined. Data are expressed as a percentage of maximal possible effect (30 s for HP and 15 s for TIT) and presented as mean ± SEM. (**A**) *—*p* = 0.03; **—*p* = 0.0011 and 0.05; ***—*p* < 0.001. (**B**) *—*p* = 0.03; **—*p* < 0.01; ***—*p* < 0.001.

To determine whether morphine causes different antinociceptive effects on male and female mice, and how it affects the development of tolerance, we performed HP, TIT, and OF tests. We observed that in the HP test, females demonstrated stronger antinociceptive responses to morphine compared to that in males (Figure 1A). Females stayed on the hot plate at least 2-times longer after morphine injection. Moreover, by day 7, male mice demonstrated the development of tolerance to a daily 15 mg/kg morphine administration (6% of MPE), while females demonstrated at least 20% of MPE by day 9 (Figure 1A). In the TIT assay, the acute response to morphine administration on day 1 was similar between males and females (Figure 1B). However, male mice demonstrated a fast development of tolerance, 6% of MPE by day 10, while females continued to respond to a daily morphine administration with more than 60% of MPE on day 10 (Figure 1B). The female’s response to morphine dropped to 5% of MPE on day 20 of morphine.

**Figure 2:**
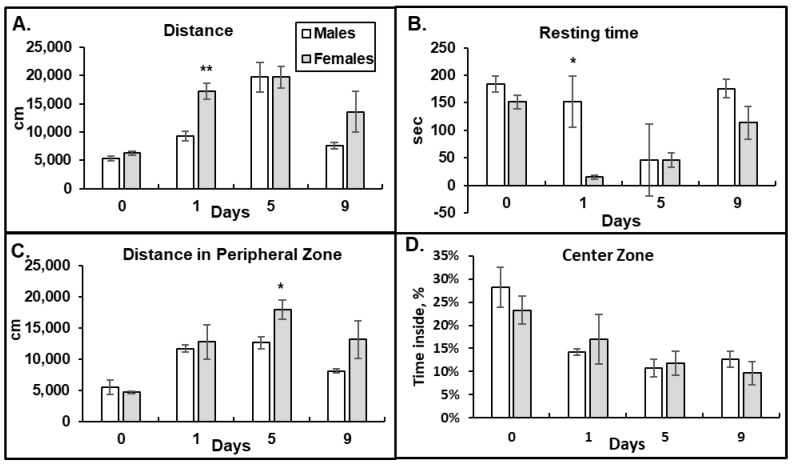
Female mice have higher acute responses to morphine in the Open Field tests than males. High doses of morphine induce anxiety in male and female mice. Distance ran in cm (**A**), resting time in seconds (**B**), distance ran in peripheral zone (**C**), and percent of time spent in the central zone of the chamber (**D**) that five male and six female mice demonstrated during 15 min interval after 15 mg/kg morphine i.p. injection. Data are expressed as mean ± SEM. (**A**) **—*p* = 0.0013. (**B**) *—*p* = 0.02. (**C**) *—*p* = 0.03.

To investigate the effect of morphine on locomotion in male and female mice, we performed the OF test. We observed that after the first morphine injection, the female mice ran longer distances with decreased resting times compared to that in males, suggesting a higher activation by morphine (day 1, Figure 2A,B). The female mice also demonstrated higher numbers of ambulatory counts of large movements, fewer bursts of small movements, and a decreased frequency of rearing breaks after acute morphine administration compared to those in the male mice. By day 5 of the morphine treatment, the male and female mice demonstrated similar patterns in the OF tests—increased running distances and decreased resting times compared to baseline, indicating similar morphine effects (day 5 vs. 0, Figure 2A,B). By day 9, the OF parameters in the male group started to return to the baseline levels faster than that in the female group, but the difference was not statistically significant (day 9, Figure 2A,B). These data suggest that female mice are more sensitive to acute morphine administration (higher HP and OF responses on day 1) and have delayed developments of tolerance by chronic morphine compared to that in male mice.

To investigate whether male and female mice experience different patterns of opioid-induced anxiety, we measured the distance the mice ran in the peripheral zone and the percent of time mice spent in the center zone of the OF chamber after morphine administration (Figure 2C,D). Avoidance of the open space in the center zone of the OF chamber indicates a higher anxiety level. We observed that after morphine administration, both the male and female mice ran longer distances in the outside area of the OF chamber and spent less time in the central area of the OF chamber (Figure 2C,D). These results suggest that morphine administration increases anxiety levels in both male and female mice. Although there were statistically significant differences in the distances female mice ran in the perimeter of the OF chamber on day 5 of morphine administration (Figure 2C, *p* = 0.03), suggesting a higher level of anxiety, the percentage of time spent in that area was similar between the male and female animals (Figure 2D). By day 9 of the 15 mg/kg morphine administration, both the male and female mice still demonstrated increased levels of anxiety compared to the baseline (day 9. Figure 2D).

### 3.2. Morphine-Induced Neuronal Degeneration

In our previous study, we demonstrated that chronic 30-day oxycodone administration increases pro-apoptotic signaling in rat brains [26]. To investigate whether chronic morphine administration induces pro-apoptotic signaling in mice brains, we performed immunohistochemical analyses of the brain slices of male and female mice to evaluate the level of Bax, activated caspase 3, and pro-survival BDNF. In addition, we performed the TUNEL assay to evaluate neuronal death. Male and female mice were injected i.p. with either 15 mg/kg morphine or saline for 30 days.

#### 3.2.1. Male Mice Have Higher Constitutive Bax Expression Than Females. Chronic Morphine Increases Bax Expression in Mouse Brains but Not the Number of Pro-Apoptotic Cells

**Figure 3:**
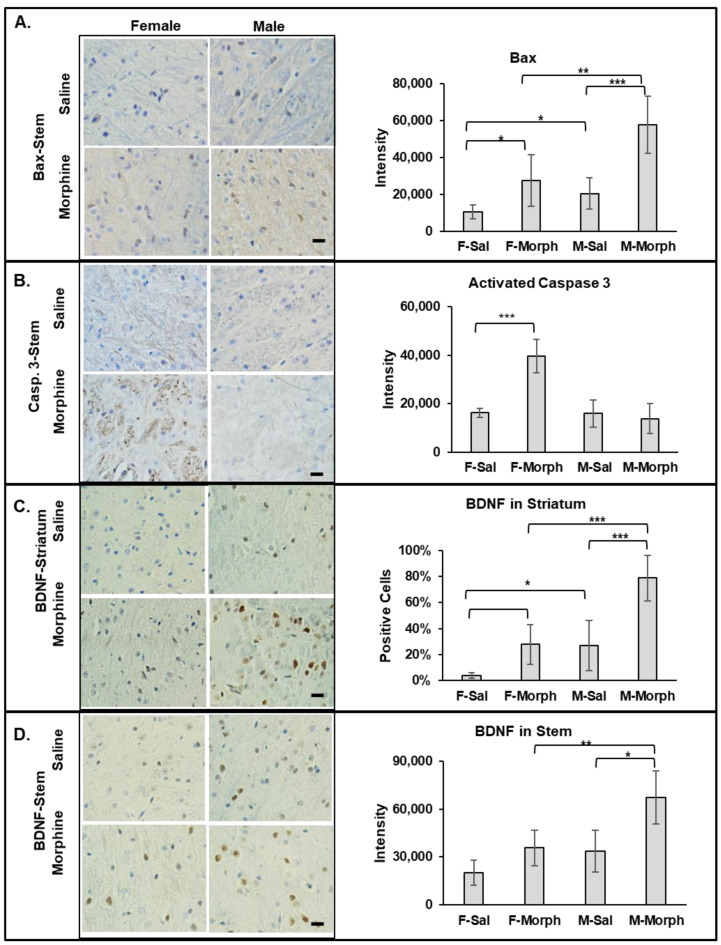
Male mice have higher constitutive Bax and BDNF levels in the brain. Morphine increases Bax and BDNF signaling in both male and female mouse brains, and activated caspase 3 only in females. Left: immunohistochemical staining of Bax (**A**), activated caspase 3 (**B**), and BDNF (**C**,**D**) expressions in male (right columns) and female (left columns) mice treated with saline (upper images) or morphine (lower images). Bars are 20 μm. Right: quantitative analysis of Bax, activated caspase 3, and BDNF using ImageJ (FIJI) software. Data are expressed as mean ± SD, *n* = 6. *—*p* < 0.05; **—*p* < 0.01; ***—*p* < 0.001.

Bax (Bcl-2-associated X protein), when activated, induces cell death via the mitochondrial apoptosis pathway. We observed that the number of Bax-positive cells and the intensity of Bax staining increased in both the striatum and stem areas in 30-day morphine-treated males and females compared to that in the corresponding saline-treated animal groups. In the female striatum, morphine almost doubled the number of Bax-positive cells from 10% ± 2% in saline- to 20% ± 7% in morphine-treated animals (mean, SD, *n* = 6; *p* = 0.007). In the male striatum, the morphine-induced increase in the number of Bax-positive cells was modest: from 22% ± 2% in saline- compared to 33% ± 9% in morphine-treated animals (mean, SD, *n* = 6; *p* = 0.08). Interestingly, the both saline- and morphine-treated male striatum tissues had higher Bax-positive cell numbers and intensity compared to the corresponding female treatment group. Similarly, in the stem, morphine increased Bax expression in both male and female tissues compared to that in the saline-treated animals, with *p* = 0.02 for saline vs. morphine in the female group and *p* = 0.0004 in the male group (Figure 3A). Both the saline- and morphine-exposed male stem tissues contained higher levels of Bax compared to that in the corresponding female tissues: *p* = 0.03 (saline female vs. male) and *p* = 0.008 (morphine female vs. male) (Figure 3A). These data suggest that neuronal cells in the male mice brain have higher constitutive Bax expression compared to that in female mice. These data also demonstrate that morphine increases the level of pro-apoptotic Bax in neuronal cells in both male and female mouse brains.

#### 3.2.2. Chronic Morphine Activates Caspase 3 in Female Brains

In our previous study, we observed that chronic oxycodone treatment induced the expression of activated caspase 3 in female rats [26]. To investigate whether morphine induces activated caspase 3 in male and female mouse brains, we performed IHC analysis on the brain slices. We observed a significant increase in activated caspase 3 signaling only in the female morphine-exposed brains (Figure 3B). Activated caspase 3 was detected in the axons of the white matter in the striatum and brain stem areas of the morphine-treated female mice. The highest staining was observed in the cerebral peduncle and medial lemniscus of the female brains. The male brain tissue did not show a significant expression of activated caspase 3, not in the saline- nor in the morphine-treated animal groups (Figure 3B). These data suggest that chronic morphine administration differentially activates caspase 3 in axons in female brains.

#### 3.2.3. Chronic Morphine Increases BDNF Expression. Male Mice Have Higher Constitutive BDNF Expression Than Females

To investigate whether morphine induces pro-survival signaling in the mouse brain, we monitored expression of brain-derived neurotrophic factor (BDNF). BDNF is a neurotransmitter modulator involved in synaptic plasticity and memory formation, neuronal growth, and survival. We observed an increase in BDNF expression in the striatum, stem (Figure 3C), and hippocampus in both male and female brains after 30 days of morphine administration. Interestingly, BDNF expression was higher in the male saline- and morphine-exposed brain tissues compared to that in the corresponding female brain tissues. These data suggest that neuronal cells in the male mouse brain have higher constitutive BDNF expression compared to that in female mice. These data also demonstrate that morphine increases the level of BDNF in neuronal cells in both male and female mouse brains.

#### 3.2.4. Male Mouse Brains Have Higher Numbers of Apoptotic Cells Than Females: Chronic Morphine Does Not Induce Neuronal Apoptosis

**Figure 4:**
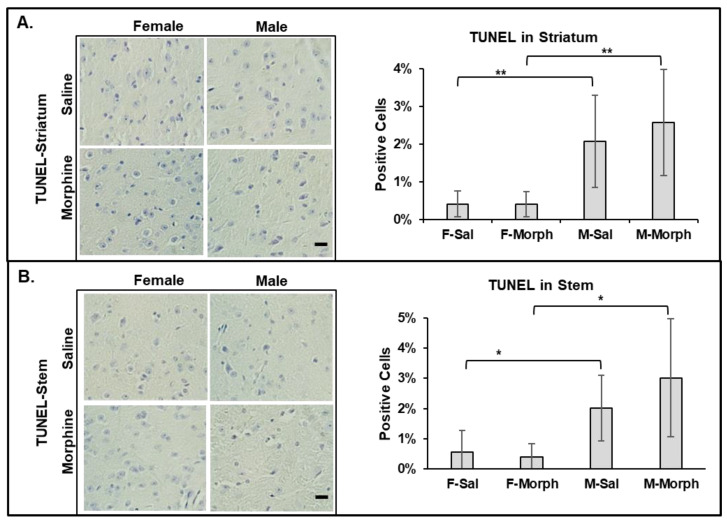
Male mouse brains contain higher numbers of pro-apoptotic cells. Morphine does not induce cell death in mouse brains. Left: representative images of TUNEL assay of striatum (**A**) and stem (**B**) brain tissues. Bars are 20 μm. Right: quantitative analysis of TUNEL-positive cells using ImageJ software. Data are expressed as mean ± SD, *n* = 6. (**A**) **—*p* = 0.009 and 0.004. (**B**) *—*p* = 0.02 and 0.01.

To investigate whether male mouse brains have higher levels of pro-apoptotic cells and whether morphine induces apoptosis in the mouse brain, we performed the TUNEL assay, which detects the level of DNA fragmentation induced by apoptosis. We observed that the male mouse brains had slightly higher numbers of apoptotic cells, even in the saline-treated group: 2% vs. 0.5% in males and females, respectively (Figure 4). Chronic morphine administration did not increase the number of TUNEL-positive cells in male nor female mouse brains (Figure 4).

To investigate whether chronic morphine treatment causes locomotor deficit in mice, we performed an RtR test. We observed no changes in the locomotor activity in male or female mice after 60 days of morphine treatment. Also, we did not observe a difference in locomotor ability between the male and female mice.

#### 3.2.5. ELISA Plasma

**Figure 5:**
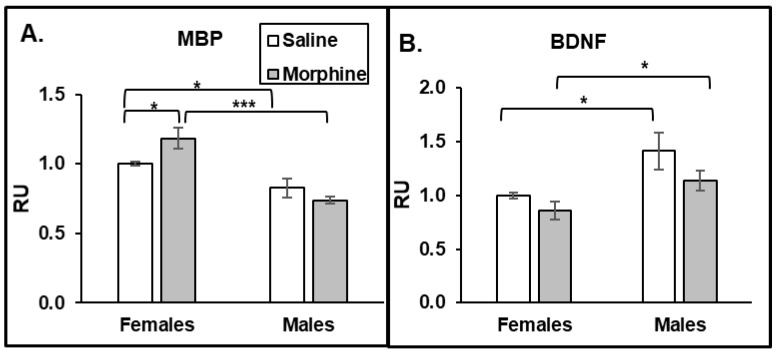
Females have higher MBP levels in plasma, but males have higher BDNF levels. Morphine increases MBP levels in female plasma. ELISA analysis of MBP (**A**) and BDNF (**B**) level in plasma of male and female mice treated with saline or morphine for 30 days. MBP and BDNF signals were normalized to the GAPDH signals in the same samples and then to the mean of the female saline value. Data expressed as mean ± SD, *n* = 6. RU, relative units. *—*p* < 0.05; ***—*p* < 0.001.

To investigate whether the increase in activated caspase 3 in axons in female mouse brains is associated with the demyelination process, we monitored the level of MBP in the plasma of the animals. We observed a morphine-induced increase in MBP levels only in plasma of female but not male mice (Figure 5A). Moreover, the baseline of plasma MBP was significantly higher in females compared to that in males. These data suggest that female mice have a higher rate of demyelination processes than males, and that this is accelerated by chronic opioid administration. One of the possible mechanisms of how male axons are protected from demyelination is that males have higher levels of BDNF compared to that in females. To verify that male mice produce more BDNF, we monitored its level in the animals’ plasma. Indeed, we observed that male plasma had higher levels of BDNF than females in both the saline and morphine groups (Figure 5B). These data suggest that male mice have a protective mechanism from demyelination by producing higher levels of BDNF.

#### 3.2.6. Testosterone Assays

**Figure 6:**
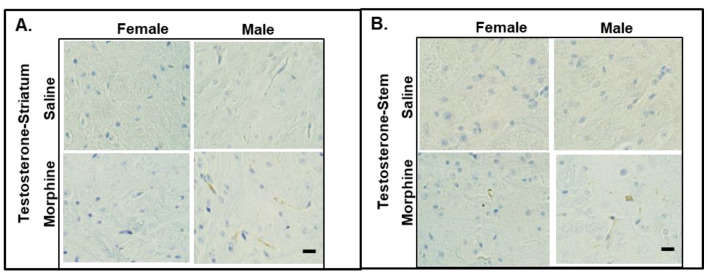
IHC staining of testosterone in male and female brains. Testosterone expression in striatum (**A**) and stem (**B**) of mice administered 15 mg/kg morphine for 30 days. Bars are 20 μm.

It was reported that testosterone regulates the CREB-BDNF transcriptional pathway, promotes myelination, and protects neuronal cells from oxidative stress and glutamate toxicity in animal models. To monitor the levels of testosterone in the mice treated with 15 mg/kg morphine for 30 days, we performed IHC staining of the brain slices and ELISA assay of the plasma samples. In the IHC analysis, we observed that the male striatum and brain stem showed testosterone staining (Figure 6). We did not detect any testosterone staining in the female brain slices.

**Figure 7:**
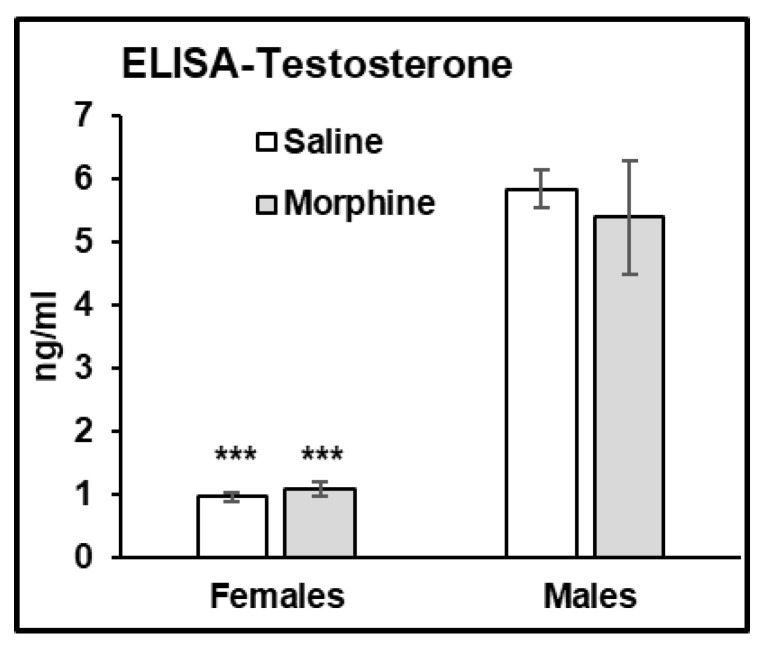
Males have higher testosterone levels in plasma. ELISA assay of testosterone concentration in saline (white bars) and morphine (gray bars) plasma samples of male and female mice administered 15 mg/kg morphine for 30 days. Data expressed as mean ± SD, *n* = 6. ***—*p* < 0.001.

In the ELISA assay, we observed that male plasma contains significantly higher levels of testosterone compared to that in female plasma (male vs. female, *p* < 0.001 in both water- and morphine-exposed samples (Figure 7)).

## 4. Discussion

Population-based studies suggest that men and women have different experiences in pain perception, drug use, drugs adverse effects, and development of neurodegenerative pathologies. Women are more prone to experience acute and chronic pain, more intense pain, and more frequent pain than men [2,5]. Women also experience a greater response to opioids [31], and females are reportedly more susceptible to drug abuse than males [2]. On another hand, males develop opioid tolerance “to a significantly greater extent” than females [7], which could lead to opioid overdose. Indeed, according to the NIDA analysis, the numbers for opioid-related overdose deaths in men for 1999–2021 are significantly higher compared to that in women [32]. In our study, we observed that female mice display higher anti-nociceptive and motion responses to acute morphine administration than male mice. These data are in agreement with reports that women demonstrate greater responses to opioids [31], probably due to a higher concentration of μ-opioid receptors (MORs) in women’s brains (rev. in [33]). We also observed that male mice develop a tolerance to chronic morphine administration faster than compared to that in female mice. This is in agreement with a study showing that male rats develop greater tolerance than females after chronic morphine administration [34]. However, our results contradict the report, showing that female mice develop tolerance to morphine faster than males [35]. This discrepancy could be due to the use of different strains of animals (C57Bl/6 vs. CD-1, our study vs. [35], respectively) and due to the different protocols used to induce morphine tolerance (15 mg/kg once daily vs. several increasing concentrations ranging from 10 to 40 or 100 mg/kg morphine three times a day, our study vs. [35], respectively).

What could be the reason for male’s and female’s different responses to opioids? It was suggested that sex hormones, differential gene expressions, metabolic pathways and rates might be the major mechanisms underlying a variety of differences between men and women [28]. A recent analysis of the neuroimaging data from two databases of human brains revealed differences in the male and female brain areas involved in processing different types of information [36]. Moreover, transcriptome analysis of the human brain identified male-biased and female-biased genes in each brain region, with more than 80% of sex-biased genes containing full or half of the androgen response element (ARE) and up to 30% of genes across different brain regions containing estrogen response element (ERE) [37]. Estrogen expression was shown to lower the pain threshold in women [7], contribute to a greater response to opioids [31], and facilitate drug abuse (rev. in [9]). Sex hormones were also shown to be neuroprotective in various degenerative pathologies [38].

In our previous study, we demonstrated that chronic 15 mg/kg oxycodone treatment for 30 days increased the extent of Bax-positive cells without inducing apoptosis in the brains of young female rats [26]. Similarly, in our current study, we observed that 15 mg/kg morphine treatment for 30 days increased the extent of Bax signaling in both male and female brains without induction of apoptosis. We also observed that male mice brains have a higher constitutive Bax expression compared to that in female mice. This correlated with a slightly higher number of pro-apoptotic cells in male brains compared to that in the female group. On the other hand, chronic morphine administration activated caspase 3 in the axons of female but not male mouse brains. This result agrees with our previous study, demonstrating not only the accumulation of activated caspase 3 in axonal bundles, but also the disorganization of axons and the loss of MBP, suggesting demyelination in female rat brains after chronic oxycodone administration [26]. We speculate that chronic opioid treatment targets axons without significantly affecting neuronal soma. In our previous study [26], we observed a decrease in MBP levels in female rat brains after chronic oxycodone administration, suggesting either faster MBP degradation or reduced levels of MBP synthesis by dendritic cells. The accumulation of MBP in the plasma of morphine-treated female mice suggests that chronic opioids induce a faster degradation of MBP. On another hand, our previous results demonstrate an increase in PDGFRα levels in the brain of female rats after chronic oxycodone administration [25], suggesting that chronic opioids also affect the maturation of dendritic cells producing MBP. In our current study, we observed that chronic morphine increased the level of MBP in female, but not in male plasma samples. Moreover, we observed that females had higher MBP levels even in saline-treated groups. These results suggest that females have more active de- or remyelinating processes in the brain that lead to an accumulation of degraded MBP in the plasma. This might be in line with the observation that women are more prone to demyelinating diseases [28] and MS. Multiple sclerosis is an autoimmune disease that damages the myelination of neurons. Women experience earlier MS onset and more frequent relapses [39]. MS in males is associated with a greater atrophy of gray matter and a worse outcome [39]. This suggests that there are different routes of inflammatory responses in male and female brains, with neuronal bodies targeted in male brains and axonal myelination processes in female brains. 

What could be a mechanism that protects neurons from demyelination in male brains? Animal studies indicate that androgens are involved in neuronal protection and the transcriptional upregulation of genes related to myelin production [40]. Particularly, testosterone has been described to protect neuronal cells from oxidative stress and glutamate toxicity [41,42], and was shown to be significantly lower in women with MS [43]. Low testosterone levels were associated with worse clinical outcomes for men with MS [44]. In our study, we observed that male mice had higher levels of testosterone in the brain and plasma samples compared to that in females, suggesting a protective mechanism against demyelination in male mice resulting from chronic opioid exposure. CREB-BDNF signaling is one of the transcriptional pathways regulated by testosterone [39,45]. It was demonstrated that BDNF-mediated activation of the cAMP/CREB pathway promotes myelination in an animal model [46], and that BDNF expression is associated with a positive neuroprotective mechanism in MS [47]. Downregulation of BDNF expression was also associated with the neuropathology of AD [48]. In our study, we observed that male mice had higher BDNF levels than females under saline and morphine treatments in both brains and plasma. This result suggests that higher constitutively expressed BDNF levels protect axons from demyelination in the male brain. In our study, we observed that chronic morphine administration increased the level of BDNF in male and female mouse brains, with male brains containing higher BDNF expressions. A human study of psychostimulants demonstrated the increase in BDNF serum levels in men to be significantly higher compared to that in women after alcohol, cocaine, and methamphetamine withdrawal [49]. In addition, clinical and preclinical studies demonstrate an association between increased BDNF serum levels and alcohol dependence [50]. These results suggest that BDNF is involved in the drug adaptation process, with a possibly higher effect on males compared to females. Considering that increasing evidence indicates that BDNF may serve a neuroprotective role in opioid use disorders [51], this might suggest a neuroprotective mechanism in male brains during chronic opioid use. Further studies are needed to identify specific pathways contributing to neuronal degeneration and neuroprotection after opioid abuse in males and females.

## Data Availability

The data that support the findings of this study are available from the authors upon reasonable request and with permission of LSU Health.
